# Chimeric NP Non Coding Regions between Type A and C Influenza Viruses Reveal Their Role in Translation Regulation

**DOI:** 10.1371/journal.pone.0109046

**Published:** 2014-09-30

**Authors:** Bernadette Crescenzo-Chaigne, Cyril Barbezange, Vianney Frigard, Damien Poulain, Sylvie van der Werf

**Affiliations:** 1 Unité de Génétique Moléculaire des Virus à ARN, Institut Pasteur, Paris, France; 2 Unité Mixte de Recherche 3569, Centre National de la Recherche Scientifique, Paris, France; 3 Université Paris Diderot Sorbonne Paris Cité, Paris, France; University of Berne, Switzerland

## Abstract

Exchange of the non coding regions of the NP segment between type A and C influenza viruses was used to demonstrate the importance not only of the proximal panhandle, but also of the initial distal panhandle strength in type specificity. Both elements were found to be compulsory to rescue infectious virus by reverse genetics systems. Interestingly, in type A influenza virus infectious context, the length of the NP segment 5′ NC region once transcribed into mRNA was found to impact its translation, and the level of produced NP protein consequently affected the level of viral genome replication.

## Introduction

Influenza viruses are members of the *Orthomyxoviridae* family and are classified into three antigenic types, A, B and C. They have a negative-polarity RNA genome segmented into eight (type A and B) or seven (type C) single-stranded molecules. Type C virus has a unique envelope glycoprotein (HEF) whereas type A and B viruses have two (HA and NA) [1]. Each genomic viral RNA (vRNA) is encapsidated by multiple nucleoproteins (NP) and associated with the polymerase complex (P) formed by three subunits named PB1, PB2 and PA for influenza A viruses and PB1, PB2 and P3 for influenza C viruses. In the nucleus of infected cells, the messenger RNAs (mRNAs) are products of a transcription process involving a cap-snatching mechanism: the mRNA synthesis is initiated with capped RNA primers that are cleaved from host cell mRNAs. Transcription into mRNA terminates 17 to 22 nucleotides (nt) upstream of the 5′ end of the genomic vRNA template at a stretch of five to seven uridine residues used as polyadenylation signal. The syntheses of the complementary RNAs (cRNAs) and of the vRNAs are primer-independent. Anti-termination occurs at the poly U sequence during the cRNA synthesis which, itself, is used as a template for the synthesis of the genomic vRNAs [1].

For each genomic vRNA, the coding region is flanked by non coding (NC) sequences. The NC region of each genomic vRNA can be divided into two parts: the conserved sequence common to all the viral segments and specific for each type, and the non conserved sequence [2]. For the 3′ and 5′ ends, respectively, the conserved sequences are 12 and 13 nt for type A and 11 and 12 nt for type C influenza viruses [3], [4], [5]. The total length of the NC sequences of each segment of influenza A and C viruses is very variable and depends on the non conserved sequence length [6]. This is particularly true for the NP segment, for which the 3′ and 5′ NC sequences are 45 and 23 nt long for type A and 29 and 102 nt long for type C influenza viruses, respectively (all numbers excluding the ATG and stop codons).

**Figure 1:**
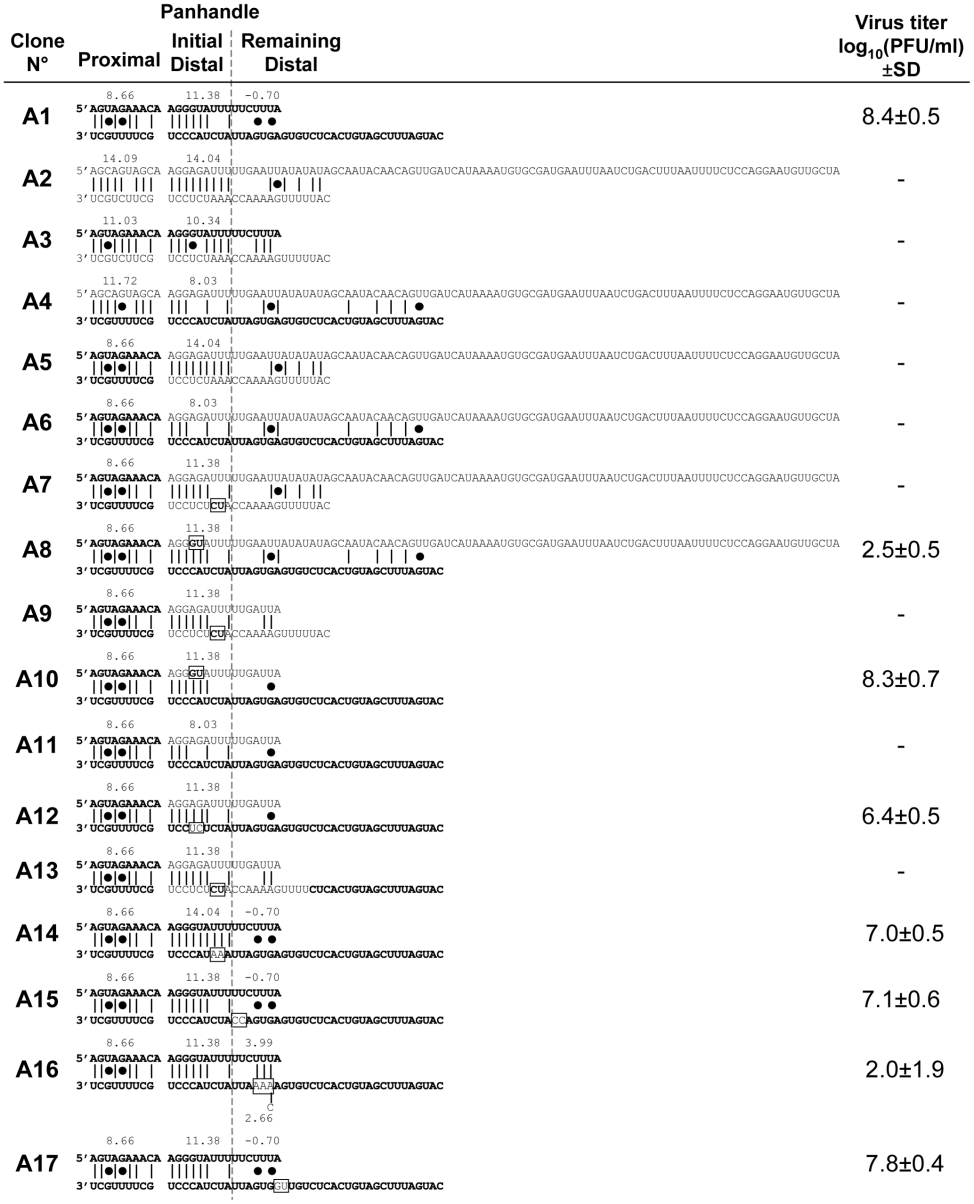
Rescue of influenza A viruses harboring type A/C substitutions and/or mutations in the NC regions of the genomic NP segment. NC region nucleotide sequences and predicted panhandle conformation for the different NP genomic segments used in type A influenza virus genetic backbone. Sequences of A/WSN/33 origin are in bold and those of C/JHB/1/66 origin are in plain. The mutations introduced by site-directed mutagenesis are shown in boxes. The sequence of both ends of each segment of each rescued virus was verified as described [9], and no mutation was detected. The energy barrier of the canonical pairs C∶G and U∶A, and of the wobble base pair G∶U (represented by a black dot) described by Vendeix et al [19] were used to calculate a score to evaluate the panhandle strengths. Titers in log_10_(PFU/ml) are the mean ± Standard Deviation (SD) of 2 to 4 independent reverse genetics experiments.

**Figure 2:**
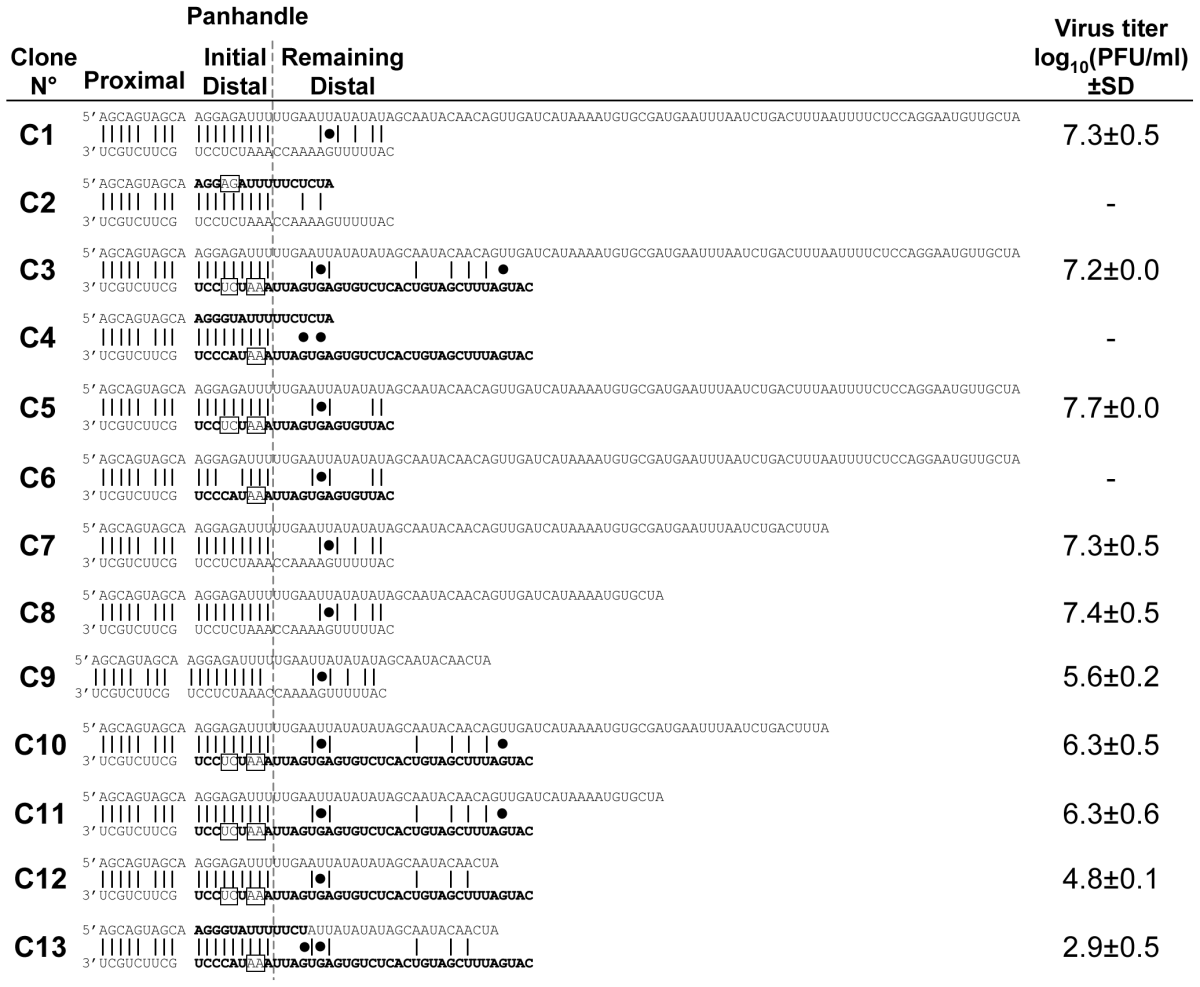
Rescue of influenza C viruses harboring type C/A substitutions and/or mutations in the NC regions of genomic NP segment. NC region nucleotide sequences and predicted panhandle conformation for the different NP genomic segments used in type C influenza virus genetic backbone. Sequences of C/JHB/1/66 origin are in plain and those of A/WSN/33 origin are in bold. The mutations introduced by site-directed mutagenesis are shown in boxes. The sequence of both ends of each segment of each rescued virus was verified as described previoulsy [9] and no mutation was detected. The energy barrier of the canonical pairs C∶G and U∶A, and of the wobble base pair G∶U (represented by a black dot) described by Vendeix et al [19] were used to calculate a score to evaluate the panhandle strengths. Titers in log_10_(PFU/ml) are the mean (± SD) of 2 to 4 independent reverse genetics experiments.

Within each genomic RNA molecule, the NC ends form secondary structures. Based on potential base-pairing, two elements were defined within the NC ends: the proximal element or region I (nt 1–9 of 3′ end and 1–10 of 5′ end) and the distal element or region II involving sequences downstream of nt 10 and 11 from the 3′ and 5′ ends respectively [7]. Two possible secondary structures formed by the 3′ and 5′ ends of each segment have been described: the ‘panhandle structure’ resulting from the base-pairing of the respective proximal and distal elements of each ends [8], [9], [10]; and the ‘corkscrew structure’ that consists of hairpin loops formed by the respective proximal elements followed by base-pairing of the distal elements [11], [12], [13], [14]. These structures are known to be critical for the virus multiplication, in particular for the transcription and the replication of the vRNAs [7]. The distal element can in fact be further divided into two sub-elements: the initial distal panhandle corresponding to the first nine nucleotides (for illustration, see A1 and C1 in Fig. 1 and 2, respectively) and the remaining distal element.

We previously studied the role in type specificity of the NC regions of the NS segment, which are quite similar in length (26 nt for the 3′ end of both types A and C, and 26 and 47 nt for the 5′ end of type A and C, respectively). We tried to rescue, by reverse genetics, type A and type C viruses with one or both heterotypic ends. We showed for the NS segment that a homotypic proximal panhandle was necessary to obtain infectious influenza A or C viruses by reverse genetics [15].

Here we report the results of a similar approach on the NP segment, which is characterized by a large difference in the length of the NC sequences between type A and C influenza viruses, i.e. 45 and 29 nt long for the 3′ end, and 23 and 102 nt long for the 5′ end, respectively [6]. Using heterotypic constructs and site-directed mutagenesis, we studied the importance of the NC sequences to rescue infectious viruses by reverse genetics. Our results supported the importance of the homotypic proximal panhandle, as seen for the NS segment, and that the length of the genomic 5′ end influences the expression of the NP protein.

## Results and Discussion

A series of recombinant viruses based on type A and C influenza virus reverse genetics systems were constructed to identify the parts in the NC regions of the NP segment that are important for type specificity. Both wild type (wt) viruses (A1 and C1, Fig. 1 and 2, respectively) were rescued with similar efficiencies to what was previously described [16], [17], [18].

In type A reverse genetics system, no virus was rescued when one or both NP segment ends were of type C (constructs A2, A3 and A4, Fig. 1). The strength of the proximal panhandle is highly affected in these constructs (energy barrier 14.09, 11.03, 11.72 for A2, A3, A4, vs 8.66 for wt A1, based on Vendeix's formulas) [19]. We previously showed for the NS segment that a homotypic proximal panhandle is compulsory to rescue influenza virus by reverse genetics [15]. For the NP segment, point mutations to restore a type A proximal panhandle were insufficient to rescue recombinant viruses (constructs A5 and A6, Fig. 1). We nonetheless hypothesized that a homotypic proximal panhandle was required and we decided to maintain it in all further NP constructs, as it was previously shown to be needed for viral polymerase binding and RNA promoter activities [10], [20], [21], [22].

Since the strength of the initial distal panhandle was also affected in A2, A3 and A4 (energy barrier 14.04, 10.34 and 8.03 respectively) and still remained affected in A5 and A6 (energy barrier 14.04 and 8.03, respectively, vs 11.38 for wt A1), point mutations were introduced to modify this strength in order to match that of wild type (constructs A7 and A8, energy barrier 11.38, Fig. 1). Only the virus with type A 3′ end was rescued (A8), but with a dramatically lower efficiency. Type C 5′end is much longer than that of type A (102 nt vs 23 nt) [6]. To test whether this might affect the recovery efficiency, deletions were introduced in both A7 and A8 constructs to generate A9 and A10, respectively (Fig. 1). Surprisingly, A9 could not be rescued. The A10 virus was rescued with the same efficiency as wt. This was not surprising, since A1 and A10 differed by only two nucleotides. The role of the initial distal panhandle strength was further verified by modifying A10 to generate constructs A11 and A12 (energy barrier 11.38, 8.03 and 11.38, Fig. 1). No virus was rescued when the strength of the initial distal panhandle was reverted to that of A6 while maintaining the short 5′ end (construct A11). On the contrary, modification of the sequence of the initial distal panhandle while maintaining the strength (A12) led to efficient rescue. These data based on type A influenza virus NP segment showed that it is possible to obtain virus by reverse genetics when a homotypic proximal panhandle and a homotypic strength of the initial distal panhandle are maintained, and that the length of the 5′ end plays an important role in the efficiency of rescue.

**Figure 3:**
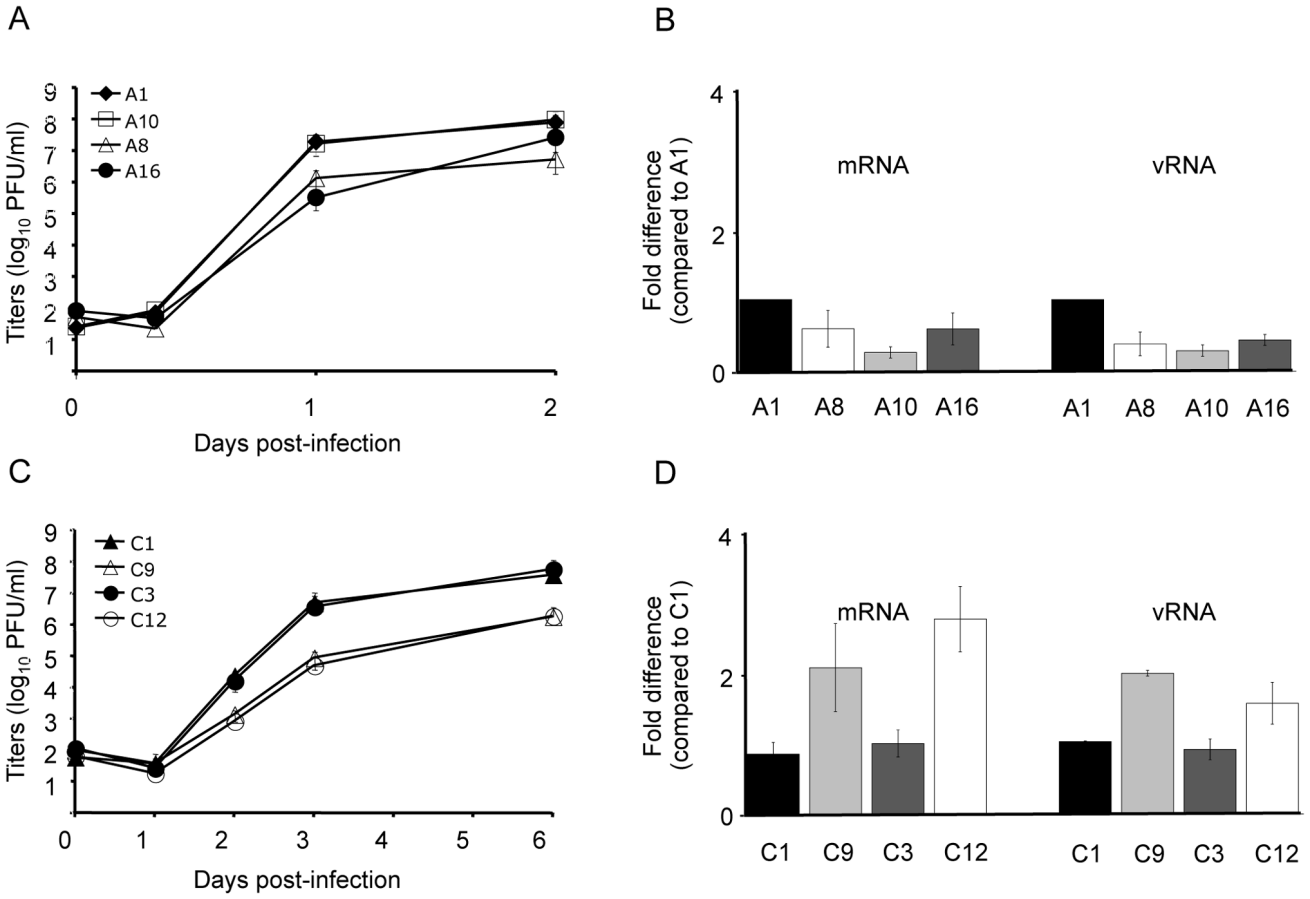
Replicative characteristics of influenza viruses with substituted or mutated NP NC regions. A) and C) Growth kinetics. MDCK cells were infected with rescued type A influenza viruses in the presence of 1 µg/ml of TPCK-trypsin at an m.o.i. of 0.001 (A). SK cells were infected with rescued type C influenza viruses in the presence of 0.5 µg/ml of TPCK-trypsin at 0.001 m.o.i (C). The supernatants were collected at the indicated times after infection and virus titers were determined by plaque assay. Results are expressed as the mean (± SD) of two independent growth kinetics. B) and D) Minireplicon assays. 293T cells were co-transfected with the expression plasmids coding for the nucleoprotein and the three subunits of the viral polymerase, and with the reverse genetics plasmid of interest, to reconstruct functional vRNP. Twenty four hours post transfection, total RNA were extracted, and viral vRNA and mRNA were quantified by RT-qPCR, along with the quantification of GAPDH mRNA as normalizer. Results are expressed as fold changes calculated compared to the respective wt construct mean using the ΔΔCt method, and are the mean (± SD) of several independent transfections (four for type A and two for type C). In order to quantify only NP mRNAs produced from the reverse genetics plasmid and not from the expression plasmid, H1N1pdm09 proteins had to be used in a type A context (B) since the primers used [48] also match PR8 NP sequence. Similarly, in the type C context (D), type A PR8 proteins were used as they were previously shown to efficiently transcribe and replicate type C pseudo-vRNA [44].

The fact that no virus was rescued for construct A9 pointed out the potential role of the 3′end in type specificity. Construct A13 differed from its parental construct A9 by an extension of the 16 nucleotides that precede the start codon in type A 3′end in order to match the length of type A 3′ end (Fig. 1). No virus was rescued and we supposed that there might be some important specific nucleotides in the type A 3′ end. To test this hypothesis, we generated constructs A14 to A17 (Fig. 1), where point mutations were introduced in the wt A1 to replace specific nucleotides by those found in type C 3′ end. The efficiency of rescue was only affected for A16, for which the strand interaction strength around the stop codon was modified (energy barrier 3.99 for A16 vs −0.7 for A1), resembling that of A9 and A13. However, all A14–A17 viruses were rescued and this could not, on its own, explain the impossibility to recover A9 and A13. Type C NP segment was demonstrated to be efficiently transcribed and replicated by type A polymerase in an artificial system based on CAT expression [6]. Although only the proximal and initial distal panhandles were described as required to initiate transcription and replication [23], minireplicon assays using the same plasmids used for reverse genetics to generate vRNA templates showed that transcription and replication levels were only slightly affected for A16 (Fig. 3B) and A13 constructs (data not shown), but were largely impaired for A9 construct (reduced by about a 100 fold compared to wt A1 construct). We speculate that impaired transcription/replication processes of the NP segment might not be the main obstacle to reverse genetics recovery and that, somehow, packaging of the NP segment could be prevented for A9 and A13. Influenza virus non conserved non coding regions are known to carry packaging signals [24],[25] and to be involved in intermolecular vRNA-vRNA interactions [26]. The influence of the sequences surrounding the stop codon thus needs to be further investigated, as several positions differ between A13 and A16.

The role of the NP segment 5′ and 3′ ends in type C virus cycle was then analyzed. Constructs with one or both type A ends were attempted (C2, C3 and C4, Fig. 2). However, based on type A results, we decided to directly maintain a homotypic type C proximal panhandle and the homotypic initial distal panhandle strength in those constructs and all the subsequent ones. Only C3 virus, with the remaining distal parts of type C 5′end and of type A 3′ end, was successfully and efficiently rescued. To confirm that the length of the 3′ end was not essential to rescue type C viruses, a deletion of 16 nt in the 3′ end of C3 was performed to generate the C5 construct, which was recovered as efficiently as C3 (Fig. 2). The importance of the initial distal panhandle strength was demonstrated by the introduction of point mutations in C5 (to generate C6). As expected, construct C6 with a weaker initial distal panhandle could not be rescued (Fig. 2).

The role of the 5′ end length on recovery efficiency was further explored by a series of deletions. On the contrary to type A virus, for which it was observed that increasing the length of the 5′ end (from 23 nt to 102 nt) impaired the recovery efficiency, one could expect that, for type C virus, the efficiency would be affected by shortening the 5′ end. A reduction of 22 or 41 nt (leading to 80 and 61 nt long 5′ ends, respectively) in C1 and C3 constructs had no effect on the recovery efficiency (constructs C7–C8 and C10–C11 for C1 and C3, respectively, Fig. 2). It was, however, affected when the 5′ end length was reduced to 42 nt (constructs C9 and C12). These results confirmed the impact of the length of the NP segment 5′ end on reverse genetics efficiency, as seen for type A virus. They are also in agreement with the variability in length that was observed for the two other sequenced strains of influenza type C viruses (C/California/78 and C/Ann Arbor/1/50), whose NP 5′ ends are 80 nt long [6]. Finally, they could explain why C4 and C2 viruses were not rescued, since the length of their 5′ end was identical to that of type A (23 nt long), so probably too short in a type C context. Extending the length in C4 5′ end by 19 nucleotides to match that of C12 5′ end allowed the recovery of recombinant virus by reverse genetics with a very low efficiency, but the virus could not be amplified from the reverse genetics supernatant (C13, Fig. 2).

After one round amplification from plaque cloning purified viruses, sequencing did not reveal any changes in the NC regions of all segments of all viable viruses compared to the plasmids used for reverse genetics. The growth abilities of the particularly interesting viruses were then evaluated by growth kinetics at low multiplicity-of-infection (m.o.i). Growth abilities of the type A virus with a long 5′ end (A8) were reduced compared to that of viruses with a short 5′ end (A1 and A10) (Fig. 3A). Conversely, type C viruses with a long 5′ end (C1 and C3) grew better than those with a short one (C9 and C12) (Fig. 3C). The sequences of the NC regions of all segments were checked for the viruses sampled at the last kinetic points, and no mutation was observed for these constructs (A1, A8, A10, C1, C3, C9, C12) compared to the stock viruses used for growth kinetics and the plasmids used for reverse genetics. Minireplicon assays revealed that transcription and replication processes were only marginally affected (Fig. 3B and 3D) and could not account for the differences observed in growth kinetics. Thus, growth kinetics confirmed the importance of the NP segment 5′ end length in influenza virus cycle. Interestingly, it seems that a short 5′ end is needed for efficient growth of type A viruses, whereas, on the contrary, type C viruses require a long 5′ end.

The growth of A16 virus was delayed compared to wt A1 at one day post infection (Fig. 3A). However, at two days post infection, the difference in titer was lower, suggesting an adaptation of A16. Sequencing of A16 NC regions at that time point revealed a mutation at position 24 in the 3′ end of the NP segment (Fig. 1). Intriguingly, this A24C modification reduced the strength of the putative interaction around the stop codon (energy barrier modified from 3.99 to 2.66). So far, it is however unclear whether or not, and, if so, how the changes introduced in the 3′ end sequence of A16 or the strength of the interaction around the stop codon affect the growth abilities of type A influenza virus.

Overall, growth abilities of A8, A16, C9 and C12 highlighted the discrepancy that might be observed between the possibility to obtain a virus by reverse genetics and the fitness of the then-obtained virus. Although A8 was not efficiently rescued by reverse genetics, its fitness was only slightly affected and it remained genetically stable. On the contrary, A16, with similar initial characteristics, seemed to have gained fitness through a single mutation.

**Figure 4:**
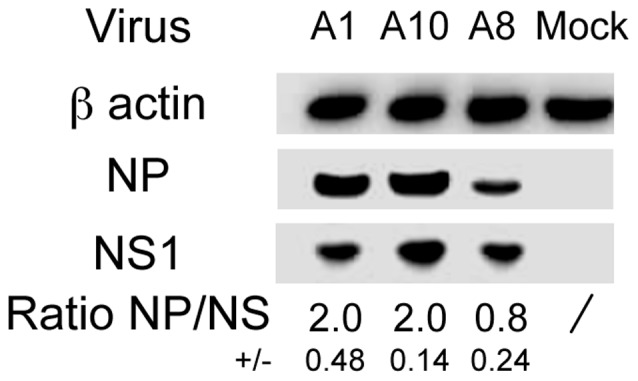
Levels of NP and NS1 proteins after infection with the rescued influenza A viruses. Six hours after MDCK infection at a high m.o.i. (2 PFU/cell), cell lysates were analyzed by Western-blot for β-actin as cellular control and viral NP and NS1 proteins. The chemiluminescence signal was acquired with a G. Box (SYNGENE, Cambridge, UK). NP and NS were analyzed on the same Western-blot. The densitometry quantification of viral protein band densities was performed for three independent replicates using GeneTools software (SYNGENE, Cambridge, UK) and the values were used to calculate the mean (± SD) NP/NS1 ratios for each virus. Due to the high levels of viral protein expression during infection, protein extracts were diluted to 1/30 in uninfected cellular extracts prior to electrophoresis (except for the β-actin detection). Results are from one representative assay out of three independent western blots.

To try and understand the impact of 5′ end length on type A influenza virus cycle, the level of expression of the NP protein was assessed at early stages of infection with A1, A10 and A8. Western-blot was performed on cellular extracts collected at six hours post infection at an m.o.i. of 2 and NP/NS1 ratios were calculated (Fig. 4). The NP protein expression was clearly reduced for the virus with a long 5′ end compared to wild type (NP/NS1 ratio of 0.8 vs 2.0 for A8 and A1, respectively). No difference was found for A10 virus compared to A1 (ratio of 2.0), indicating that the long 5′ end is responsible for the lower expression of the NP protein for A8. The mRNA levels were relatively similar between the three viruses (mean Cp values = 19.97±0.87, 19.47±0.48, 20.65±0.47 for A1, A10 and A8, respectively), suggesting that translation, rather than transcription, could be mainly affected. The 5′ end of NP viral mRNA is entirely complementary to the 3′ end of the genomic vRNA, but the viral mRNA 3′ end includes only the complementary to the non conserved NC sequence up to the polyU signal in the genomic vRNA 5′ end. Consequently, whereas the NP mRNA sequences are almost identical for A1 and A10 (with only a two nucleotide difference in the sequence between the stop codon and the polyA, and no difference in the NP protein expression levels), the NP mRNA of A8 is characterized by a longer 3′ untranslated region (UTR). It was shown that the 3′UTR of cellular mRNAs has a critical role in gene expression regulation [27]. The mRNA 3′UTR could indeed regulate every steps of translation (initiation, elongation and termination) through different mechanisms involving RNA-binding proteins, micro-RNAs or the ‘closed-loop’ structure formed by the mRNA 5′ and 3′ UTRs via the eIF4 translation initiation factors [28], [29]. Several viral families were reported to regulate the translation of their own mRNAs by such mechanisms at either the initiation (Dengue virus [30], [31]; coxackievirus [32]), the elongation (Tobacco mosaic virus [33]) or the termination (Hepatitis C virus [34]) step. We thus postulate that, in a type A influenza virus context, a long 3′UTR in the NP mRNA, or specific sequence within such long 3′UTR, could affect the level or the speed of translation through interactions with yet unknown cellular factors.

Several functions of the NP protein of influenza viruses have been described. Among them, NP would play a major role in the replication of the virus genome. More specifically, NP would be involved in the switch from capped RNA-primed transcription (protein expression) to primer-independent replication (copy of the genome). Since negative-sense RNA molecules cannot be synthesized directly from negative-sense RNAs, full-length positive-sense replicative intermediates complementary to each segment, namely cRNAs, are required as templates in influenza virus replication. The synthesis of mRNAs and cRNAs by the viral polymerase thus uses the same genomic vRNA molecules as templates, but with different initiation and termination mechanisms [35]. Different models for the role of NP in this switch from primer-dependent transcription to primer-independent replication have been proposed. The switch was first attributed to the RNA-binding properties of NP, either by stabilizing the nascent cRNA and vRNA transcripts through encapsidation [36] or by altering the RNA promoter structure [37]. However recent studies showed that changes in the equilibrium between transcription and replication could be determined by the interaction of NP with the components of the viral polymerase [38], [39].

**Figure 5:**
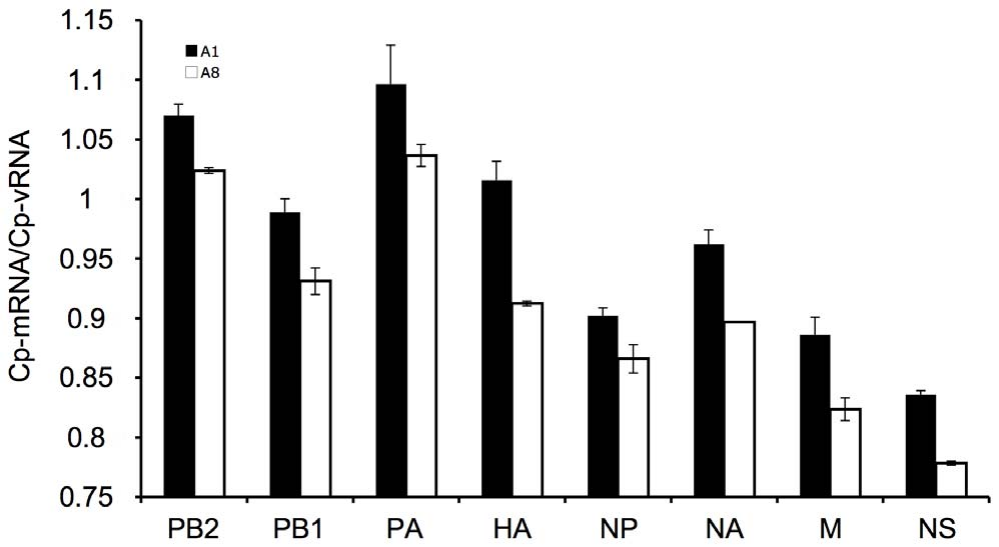
Quantification of vRNA and mRNA levels in single-cycle infections. Six hours after MDCK infection with rescued influenza A1 (black bars) or A8 (white bars) viruses at a high m.o.i. (2 PFU/cell), viral vRNA and mRNA levels for each segment were evaluated by specific two-step RT-qPCRs previously described [48]. Results are expressed as the Cp-mRNA/Cp-vRNA ratios and are the mean (± SD) of two independent infections, with six replicate points of qPCR being performed on each sample.

As a consequence of this role of NP, it can be anticipated that a lower level of NP protein for A8 virus compared to wt A1 could delay the replication. Such a delay was evaluated by comparing the levels of mRNAs and vRNAs for all the segments of these two viruses at the early stages of infection (6 hours post infection at an m.o.i of 2). The crossing-point (Cp) values obtained for each kind of RNA were combined to calculate a ratio Cp-mRNA/Cp-vRNA and these ratios were compared between the two viruses. As expected according to our hypothesis, a moderate but consistent reduction of the Cp-mRNA/Cp-vRNA ratio was observed for all segments of A8 (Fig. 5). This reduced Cp-mRNA/Cp-vRNA ratio could be attributed to a lower level of vRNA production for A8 compared to wt A1 or to a slight delay in the shift from primer-dependent transcription to primer-independent replication. These results confirmed that the consequence of a lower level of NP observed by western blot for A8 was a reduction or delay in the genome replication; and this provided a convincing explanation for the slightly impaired growth abilities of A8 virus compared to wt A1 (Fig. 3A).

## Conclusions

Using an original approach that aimed at exchanging the NC regions of influenza virus type A by those of type C and reciprocally, we showed the importance of the proximal panhandle and of the strength of the initial distal panhandle in type specificity for the NP segment. Without respecting the backbone type for these structures, no infectious virus could be rescued by reverse genetics in both type A and C systems. This confirmed the results we previously obtained with the NS segment [15], at least for the proximal panhandle, but the role of the initial distal panhandle for the NP segment highlighted the differences that exist between the different genomic segments. A similar approach on the other segments would undoubtedly reveal more intriguing features of influenza virus non coding regions.

Our results in an influenza virus infectious context also showed that the length of the 5′ NC region of type A NP segment seems to be involved in the translation regulation at the mRNA level and that the amount of NP produced subsequently affects the replication of the genome. We hypothesize that the lower growth abilities observed for type C viruses with a short 5′end (C9 and C12, Fig. 3C) were also a consequence of less NP protein due to a slightly lower level of or to a slightly slower translation. It is unclear, however, how the length would have opposite effects in type A and C influenza viruses, but this could represent another example of the numerous differences that have been described between these two influenza virus types [40], [41], [42], [43].

## Materials and Methods

### Plasmids

Plasmids pHMG-PB1, -PB2, -PA/3 and –NP, which express the PB1, PB2, PA/3 and NP proteins, respectively, of influenza virus A/Puerto Rico/8/34 (PR8) or C/Johannesburg/1/66 (C/JHB/1/66) under the control of the hydroxymethylglutaryl coenzyme A reductase (HMG) have been described previously [44], [45]. PB1, PB2, PA and NP sequences of A/Paris/2590/2009 (H1N1pdm09) were subcloned in pHMG vector.

The series of eight or seven plasmids containing the sequences of the genomic segments of A/WSN/33 or C/JHB/1/66 viruses under the control of human RNA polymerase I promoter and upstream of hepatitis Delta virus ribozyme sequence have been described previously [16], [17]. The modified heterotypic NP plasmids were constructed by PCR using primers containing the total 3′ or 5′ NC sequence of the respective NP segment, followed by cloning. Point mutations in the NP NC regions were introduced by site-directed mutagenesis, using Quikchange II Site-Directed mutagenesis kit (Agilent) according to the manufacturer's instructions. The sequences of the primers will be provided upon request. All plasmids were verified by sequencing using a Big Dye terminator sequencing kit and an automated sequencer (Perkin-Elmer).

### Cells

Madin-Darby canine kidney cells (MDCK) cells, human embryonic kidney 293T cells and human skin melanoma cells (SK93/2) [46] were cultured in DMEM supplemented with 5%, 10% and 10% fetal calf serum (FCS), respectively. All cells were grown at 37°C with 5% CO_2_.

### Production of recombinant viruses by reverse genetics

Twelve or eleven plasmid based reverse genetics systems were used to produce the recombinant type A (A/WSN/33) and type C (C/JHB/1/66) influenza viruses, and were adapted from previously described procedures [16], [17], [18]. Briefly, using 35 mm plates and DMEM supplemented with 10% FCS, co-cultures of 293T (4×10^5^/well) and MDCK cells (3×10^5^/well) for type A viruses and 293T cells (6×10^5^/well) on poly L-lysin plates for type C viruses, were transfected with the four plasmids driving the expression of the homotypic NP and polymerase proteins, together with the eight (for type A) or seven (for type C) plasmids that direct the synthesis of the vRNA templates, using 0.5 µg of each plasmid and 18 or 24 µl of FUGENE HD (Roche) for type A or type C reverse genetics, respectively. The DNA and the transfection reagent were first mixed, then incubated at room temperature for 15 min, and finally added to the cells and incubated at 35°C or 33°C for type A and type C viruses, respectively. Sixteen hours later, the DNA-transfection reagent was removed, the cells were washed twice in DMEM, and 2 mL (type A) or 6 mL (type C) of DMEM containing 0.5 µg/ml or 0.25 µg/mL of L-1-tosylamido-2-phenyl chloromethyl ketone treated trypsin (TPCK-trypsin, from Worthington), respectively, were added. The cells were incubated at 35°C or 33°C for type A or type C, respectively. Three or ten days post transfection for the type A or C, respectively, supernatants were collected for virus titration in a standard plaque assay as described below.

### Virus cloning, amplification and growth kinetics

Viruses rescued by reverse genetics were titrated and plaque-purified by plaque assay using an agarose overlay. The plaque assay protocols slightly differed for type A and C viruses and have been described previously [16], [47]. Briefly, MDCK cells were used for type A viruses and MDCK cells supplemented with bovine brain gangliosides (BBG) were used for type C viruses. The agarose overlay contained complete DMEM with additional TPCK-trypsin at 1 or 0.5 µg/ml for type A or C, respectively. Finally, overlaid cells were incubated at 35°C for 3 days or at 33°C for 5 days for type A or C, respectively.

Type A or C plaque-purified viruses were then amplified on MDCK or SK cells, respectively. Growth kinetics were performed at an m.o.i. of 0.001 on MDCK or SK cells for type A or C viruses, respectively. All these infections used DMEM supplemented with 1 or 0.5 µg/ml of TPCK-trypsin for type A or C viruses, respectively. Viral titers in the supernatant were determined at the different indicated time points post infection by plaque assay, as described aboved.

### Sequencing of 3′ and 5′ NC ends of the genomic RNA segments

The sequence of the 3′ and 5′ NC regions of all genomic RNA segments were verified for all rescued viruses. Viral genomic RNA was extracted using the QIAamp Viral RNA Mini kit (Qiagen) from 140 µl of culture supernatant following the manufacturer's recommendations. The RNA was eluted in 60 µl of RNase-free water, and the 3′ and 5′ NC regions were amplified by RT-PCR, as previously described [9]. After purification, the PCR products were sequenced using a Big Dye terminator sequencing kit and an automated sequencer (Perkin Elmer). All sequences of the primers are available from the authors upon request.

### *In vitro* transcription and replication

Minireplicon assays were performed in 293T cells transfected using 10 µl FUGENE HD with 0.5 µg of PB1, PB2, PA and 1 µg of NP type A H1N1pdm09 or PR8 expression protein plasmids for type A or type C, respectively, in presence of 0.1 ng of NP constructs. Twenty four hours post-transfection, total RNAs were extracted, reverse transcribed using the 3′ universal primer for the vRNAs (5′AGGGCTCTTCGGCCAGCRAAAGCAGG [48] or 5′AGCAGAAGCAG for type A and type C influenza virus, respectively), or an oligo dT for the mRNAs. The cDNAs were used as templates for quantitative PCR (qPCR) using a LightCycler 480 (Roche) PCR machine. Primers designed by Marsh et al. [48] and Matsuzaki et al. [49] were used for the detection of the type A and type C NP segment, respectively. As normalizer, one-step qRT-PCR targeting the GAPDH sequences was performed [50].

### Western blot

MDCK cells infected at an m.o.i. of 2 with the studied influenza A viruses were collected at six hours post-infection, solubilized in LDS sample buffer (Invitrogen) complemented with β-mercaptoethanol, and separated by 4–12% polyacrylamide gel electrophoresis (NUPAGE Invitrogen) followed by western blotting. An anti β-actin antibody (Abcam, Cambridge, UK) was used to control loadings. An in-house produced rabbit anti-influenza virus PR8 polyclonal antibody was used to detect NP [51]. The anti-NS rabbit polyclonal antibody was a kind gift from D. Marc (INRA, Tours, France).

### Single-cycle growth kinetics and detection of intracellular viral RNAs

MDCK cells were infected at an m.o.i. of 2 with the studied influenza A viruses. At six hours post-infection, total RNAs were extracted, and reverse transcriptions and qPCR were performed as described above. Separate qPCRs were performed with segment-specific primers [48] using a LightCycler 480 (Roche) PCR machine. The relative amounts of vRNAs and mRNAs were estimated by calculating the ratio mRNA/vRNA using the crossing point values (Cp).
